# Future perspectives in melanoma research. *Meeting report from the "Melanoma Research: a bridge Naples-USA. Naples, December 6^th^-7 ^th^2010"*

**DOI:** 10.1186/1479-5876-9-32

**Published:** 2011-03-26

**Authors:** Paolo A Ascierto, Eleonora De Maio, Stefano Bertuzzi, Giuseppe Palmieri, Ruth Halaban, Mary Hendrix, Mohamed Kashani-sabet, Soldano Ferrone, Ena Wang, Alistair Cochran, Licia Rivoltini, Peter P Lee, Bernard A Fox, John M Kirkwood, Claudio Dansky Ullmann, Frederic F Lehmann, Mario Sznol, Douglas J Schwartzentruber, Michele Maio, Keith Flaherty, Jerome Galon, Antoni Ribas, James Yang, David F Stroncek, Nicola Mozzillo, Franco M Marincola

**Affiliations:** 1 Department of Melanoma, Sarcoma, and Head and Neck Disease, Istituto Nazionale Tumori Fondazione Pascale, Naples, Italy; 2 Office of the Director, Office of Science Policy Analysis, Office of Science Policy, National Institutes of Health, Bethesda, MD, USA; 3 Unit of Cancer Genetics, Institute of Biomolecular Chemistry, National Research Council (CNR), Sassari, Italy; 4 Department of Dermatology, Yale University School of Medicine, New Haven, CT, USA; 5 Robert H. Lurie Comprehensive Cancer Center, Northwestern University Feinberg School of Medicine, Chicago, IL, USA; 6 Center for Melanoma Research and Treatment, California Pacific Medical Center Research Institute, San Francisco, CA, USA; 7 University of Pittsburgh Cancer Institute, Pittsburgh, PA, USA; 8 Infectious Diseases and Immunogenetics Section (IDIS), Department of Transfusion Medicine, Clinical Center and Center for Human Immunology (CHI), NIH, Bethesda, Maryland, USA; 9 David Geffen School of Medicine at UCLA, Los Angeles, CA, USA; 10 Unit of Immunotherapy of Human Tumors, IRCCS Foundation, Istituto Nazionale Tumori, Milan, Italy; 11 Department of Medicine, Stanford University, Stanford, CA, USA; 12 Laboratory of Molecular and Tumor Immunology, Robert W. Franz Cancer Research Center, Earle A. Chiles Research Institute, Providence Portland Medical Center, Portland, Oregon, USA; 13 Department of Molecular Microbiology and Immunology, Oregon Health and Science University, Portland, Oregon, USA; 14 Department of Medicine, Division of Hematology/Oncology, University of Pittsburgh Cancer Institute, and Melanoma Program, Pittsburgh Cancer Institute, Pittsburgh, PA, USA; 15 Clinical Investigations Branch, Cancer Therapy Evaluation Program DCTD, National Cancer Institute, Bethesda, MD, USA; 16 Cancer Immunotherapeutics Business Unit, GlaxoSmithKline Biologicals, Rixensart, Belgium; 17 Yale Cancer Center, New Haven, CT, USA; 18 Indiana University Health Goshen Center for Cancer Care, Goshen, IN, USA; 19 Medical Oncology and Immunotherapy, Department of Oncology, University, Hospital of Siena, Istituto Toscano Tumori, Siena, Italy; 20 Massachusetts General Hospital Cancer Center, Boston, MA, USA; 21 Inserm Group Leader, Inserm U872, Team 15, Cordeliers Research Center, Paris, France; 22 Department of Medicine, Jonsson Comprehensive Cancer Center, UCLA, Los Angeles, California, USA; 23 Surgical Brach, NIH, Bethesda, MA, USA

## Abstract

Progress in understanding the molecular basis of melanoma has made possible the identification of molecular targets with important implications in clinical practice. In fact, new therapeutic approaches are emerging from basic science and it will be important to implement their rapid translation into clinical practice by active clinical investigation.

The first meeting of Melanoma Research: a bridge Naples-USA, organized by Paolo A. Ascierto (INT, Naples, Italy) and Francesco Marincola (NIH, Bethesda, USA) took place in Naples, on 6-7 December 2010.

This international congress gathered more than 30 international and Italian faculty members and was focused on recent advances in melanoma molecular biology, immunology and therapy, and created an interactive discussion across Institutions belonging to Government, Academy and Pharmaceutical Industry, in order to stimulate new approaches in basic, translational and clinical research. Four topics of discussion were identified: New pathways in Melanoma, Biomarkers, Clinical Trials and New Molecules and Strategies.

## Introduction

Before reporting the interesting data that emerged from the debate [1,2] there is merit in mentioning discussions about the impact of biomedical research on health and wealth. In fact, the first topic addressed was the impact of biomedical research on health and wealth in USA.

Over the past 30 years, total national spending on health care has more than doubled as a share of Gross Domestic Product (GDP). Health Care Expenditure Projections suggest that health care costs will continue to account for a steadily growing share of GDP, reaching 41 percent by 2060 and 49 percent by 2082. Biomedical research is, indirectly, one of the major drivers of health care costs and at least 50% of this increased cost is attributable to it. Broadly, Federal funds for Research and Development compete with other priorities in the Federal budget and their investment is sometimes criticized for lack of results or use in non-essential projects.

Therefore it was necessary to develop a strategy to document the outcomes of science investments to the public and to ensure that resources are allocated wisely. The STAR METRICS (Science and Technology for America's Reinvestment: Measuring the Effect of Research on Innovation, Competitiveness and Science) project is a partnership between science agencies and research institutions and promises to document with solid evidence the returns that the USA is obtaining from its investment in research and development. The program is structured in two phases. The first phase will develop uniform, auditable and standardized measures of the impact of science spending on job creation, using data from research institutions' existing database records. The second phase will measure the impact of Federal science investment on four key areas: scientific knowledge (using metrics such as publications and citations), social outcomes (e.g. health outcomes measures and environmental impact factors), workforce outcomes (e.g. student mobility and employment), and economic growth (e.g. tracing patents, new company start-ups and other measures). Data for the program will come from research institutions that volunteer to participate and the federal agencies that fund them. Information will be gathered from the universities in a highly automated way, with minimal or no burden for the scientists and the university administration. This initiative provides a new way to measure the impact of federally funded research, so that the public will have an informed picture of the benefits obtained from the money spent [3].

## New Pathways In Melanoma

Genetic alterations, somatic or inherited, play a role in the pathogenesis of melanoma. The relevance of identifying genetic variants, their roles in critical pathways and in development of aggressive phenotypes in order to find new targets for melanoma therapy have been discussed.

Genetic variants in melanoma susceptibility and pathogenesis lead to different molecular subsets of melanomas. Immunohistochemical and mutational analysis showed that inactivation and impairments of the p16CDKN2A gene are present at steadily increasing rates as lesions move from primary melanoma to melanoma metastases, correlating with progression of disease and cell proliferation. Relative risk of carrying a CDKN2A mutation for melanoma patients was demonstrated to significantly increase with the presence of familial occurrence of melanoma (likelihood of CDKN2A germline mutations increases according to number of affected members in the family), multiple primary melanomas, and early age of onset. Based on such clinical predictors for germline mutations, standardized criteria have been elaborated to select putative carriers of mutations, who are at risk of developing not only melanoma but also pancreatic carcinoma. In Italy, the prevalence of CDKN2A mutations may vary widely among patients with different geographical origins. In particular, a higher frequency of CDKN2A germline mutations has been observed in patients from Northern Italy in comparison to those from Southern Italy. Mutations in CDKN2A, CDKN2B, and CDK4 genes are reported to be absent in Sardinian patients; in such a population, germline mutations in BRCA2 gene and multiple MC1R variants contribute to melanoma susceptibility. More generally, MC1R variants seem to increase melanoma risk in families with CDKN2A mutations and CDKN2A mutation carriers with MC1R variants have a statistically significant lower median age at diagnosis. Recently, a synergistic relationship between germline MC1R variants and somatic BRAF mutations has been suggested, whereby MC1R variant genotypes seem to confer a significantly increased risk of developing BRAF-mutant melanoma in skin not damaged by sunlight. It has been hypothesized that intermittent sun exposure may indirectly induce BRAF mutations through the impairment of MC1R and an increased production of free radicals. Since this correlation has not been confirmed in Australia, one could again speculate that differences in patients' geographical origins and/or the genetic backgrounds of patient populations may play an important role in determining such geographical discrepancies.

Additional information about melanoma susceptibility could be obtained from genome-wide association studies (GWAS) which aim to identify common genetic variants contributing to melanoma risk. Worth mentioning is the recently-described association between the CDKN2A locus and nevus formation as well as susceptibility to melanoma alone or melanoma and basal cell carcinoma. Although several other genes have been associated with the melanoma risk only (MC1R) or with susceptibility to melanoma and basal cell carcinoma (TYR, ASIP, and TYRP1 - which represent the major determinants of hair and skin pigmentation), their role in melanoma development remains unclear. On the basis of this evidence, a complex connection of molecular mechanisms has been implicated in melanomagenesis, raising the need to address alternative genetic progression models rather than the multi-step linear models used so far. In fact, the different molecular mechanisms may have separate roles or cooperate during all evolutionary phases of melanocytic tumorigenesis: not one but several roads lead to melanoma. Focusing mainly on BRAF, evidence has been provided suggesting the lack of close correlation in pathogenetic mutations between primary tumor and metastasis from the same patients. This could be explained by the presence of polyclonality in the primary tumor, similar to the recent finding for melanocytic nevi and in line with the recent stem cells progression model. Therefore, different molecular mechanisms generate different subsets of melanoma patients with distinct aggressiveness, clinical behavior, and response to therapy. In this sense, characterization of molecular mechanisms could contribute to better classification of the different subsets of melanoma patients and might be useful to optimally managing melanoma patients with differencing prognosis as well as to better address the most effective therapy for different melanoma subsets.

Along this line, results from sequencing the melanoma transcriptome and exome have generated new insights into melanoma biology. High-throughput sequencing by Illumina GA of tumor cDNA and exons of about 16,000 genes captured by NimbleGen arrays from tumor DNA and matching germline DNA isolated from circulating lymphocytes or skin cells, provide an unprecedented overview of novel somatic and inherited mutations in melanoma. The current experience indicates that the number of somatic variants is highly variable depending on the type of melanoma. The highest number of somatic variants was observed in a desmoplastic melanoma excised from the forehead. High prevalence of UV signature C > T mutations was observed in melanomas from sun-exposed lesions. In the absence of frequent novel recurrent mutations in specific genes such as in BRAF and NRAS, the bioinformatic analysis revealed mutations in novel genes belonging to signaling pathways involved in cell cycle control, proliferation, cell-cell interaction, cell-stroma interaction, adhesion, movement and spreading, or genes that can promote drug resistances. The focus is currently on identifying mutations in functional groups involved in activities characteristic of the malignant phenotype with a priority on kinases or other enzymes with potential to be therapeutic targets.

Exposure to an embryonic stem cell (hESC) microenvironment reprograms the metastatic phenotype of aggressive melanoma cells resulting in the re-expression of melanocyte-specific markers and a reduction in invasive potential. Regulation of the re-emergence of Nodal signaling in tumor cells is one of the possible molecular mechanisms underlying reversion of the metastatic phenotype. To better characterize the role of Nodal, the expression of key components of the Nodal signaling pathway was examined in human normal, neoplastic and hESC types. Given the significant observation that like hESCs, cancer cells express Nodal, although unlike hESCs, they do not express Lefty (Nodal's inhibitor), it was hypothesized that hESC-derived Lefty and possibly other tumor-suppressive factors found in hESC-conditioned matrices (CMTX), reprogram metastatic melanoma cells by inhibiting Nodal signaling. Further analysis showed that exposure to hESC CMTX down-regulates Nodal expression in metastatic melanoma cells and that this effect is reversible over time. Moreover, knock down of Lefty in hESC CMTX results in the up-regulation of Nodal. It has also been shown that another protein is involved in Nodal expression regulation. Indeed, the Nodal gene has a node specific enhancer (NDE) that is active in aggressive melanoma cells in a Notch-dependent manner. In particular, Notch4 is specifically required for expression of Nodal in aggressive cells and plays a vital role both in the balance of cell growth and in the regulation of the aggressive phenotype. Inhibition of Notch4 signaling blocks vasculogenic mimicry and anchorage independent growth. These data regarding Nodal signaling and its regulation offer a potential molecular target for melanoma therapy. In future Nodal may be regarded as a prognostic factor since Nodal expression is associated with vertical growth in dysplastic nevi; melanoma in situ showed lower levels of Nodal than deep melanoma and metastatic melanomas. In patients with a previous history of melanoma there was a positive correlation between high Nodal expressing nevi and melanoma Breslow depth.

Finally, it will be important to identify biomarkers that in the future may become a target for molecular therapy of melanoma. One possible approach is cDNA microarray analysis, which has enabled the identification of putative melanoma biomarkers by virtue of their differential expression in distinct phases of melanoma progression [4]. Application of cDNA microarray analysis has, for example, led to the development of multi-marker diagnostic [5] and prognostic [6,7] assays that are nearing clinical application. More recently, this approach has led to the discovery that PHIP, involved in the IGF pathway, represents a positive prognostic factor for melanomas that overexpress it. Overall, new results are emerging about the identification of progression biomarkers that can predict the ability of melanoma to metastasize to lymph nodes or to distant sites. These biomarkers can be used to identify patients at higher risk of relapse or death who may be candidates for sentinel lymph node biopsy or adjuvant therapy and may also represent possible novel targets for the molecular therapy of melanoma.

## Biomarkers In Melanoma

The hypothesis that cancer is driven by tumor-initiating cells (known as cancer stem cells) has recently attracted attention, owing to the promise of a novel cellular target for the treatment of solid malignancies. Furthermore, it seems that tumor-initiating cells might be resistant to many conventional cancer therapies, which might explain the limitations of these agents in curing human malignancies. For this reason, there is a need to find markers that serve to identify tumor-initiating cells and thus facilitate development of therapeutic strategies to target these cells. ABCB5, an ATP-binding cassette (ABC) family member, in combination with aldehyde dehydrogenase1A1 identifies melanoma initiating cells, since these cells in low numbers can induce tumors in immunodeficient mice. These cells are sensitive to cyclopamine, an inhibitor of the hedgehog signaling pathway, but are resistant to paclitaxel. Melanoma initiating cells are sensitive to BRAF inhibitors. Their antiproliferative activity can be enhanced by monoclonal antibodies specific for the membrane bound chondroitin sulphate protidoglycan 4 (CSPG4), a tumor antigen which plays an important role in the biology of malignant cells.

The efforts in biomarker identification relevant to immune mediated tumor rejection, mechanisms of therapeutic intervention and prediction of clinical outcome have been advanced by application of high throughput molecular technologies. Using minimally invasive needle biopsies, the same lesion can be monitored at the whole transcriptome level at different stages along the natural history of melanoma or during therapeutic intervention. Studies based on gene expression profiling in identical lesions before and after different types of immune therapy demonstrated a unique molecular signature in the tumor microenvironment when rejection occurs. Among these signature genes, IRF1 (IFN regulatory factor 1) up regulation has been the key immune modulator associated with responsiveness not only in melanoma but also in the response of genital warts to imiquimod, carcinoid tumors to IFN-α and CML to IFN-α. High dose IL-2 induced melanoma regression is associated with up regulation of NKGC5, T cell receptor alpha chain and HLA II related transcripts. Those genes have also been reported in association with acute rejection of renal allografts. The best self controlled melanoma study is the analysis of patients with mixed treatment responses. With identical genetic make up and immune pressure, the differences between the phenotypes of separate and distinct lesions emphasize the importance of tumor microenvironment. This study revealed that antigen presentation machinery in responsive metastases was significantly enhanced compare with progressive lesions. In the mechanism of rejection study, local applications of the TLR-7 agonist imiquimod for the treatment of basal cell cancer revealed earliest upregulated cytokine receptor CXCR3, a ligand for IP-10 and monokine induced by IFN (MIG/CXCL9), suggesting its early involvement in the crosstalk leading to migration and activation of monocytes and lymphocytes. With regard to prediction of immune responsiveness and survival, Wang identified 100 genes with significant differential expression by TILs from 13 complete responders and 40 non-responders. However, when the tumors that were the source of the TILs were studied, no clear predictors of their phenotype could be identified, suggesting that response or progression could result from intrinsic genetics of the patient rather than the specific genetics of the tumor. In conclusion, clinical outcomes of patients treated by immune therapy are determined by multiple factors that may be redundant, synergistic or contrasting. To fully understand each component's contribution to the outcome, a system biology approach should be applied.

Emerging molecular genetic techniques will increasingly be used to supplement skilled morphological tissue assessment, to optimize management of melanoma patients by increasing accuracy of diagnosis, permitting individualized prognostication and guiding optimal therapy. Fluorescence in Situ Hybridization (FISH), Comparative Genomic Hybridization (CGH), Gene microarrays (gene signatures) and Gene sequencing are techniques that can supplement the histological diagnosis of non classical melanocytic lesions such as borderline lesions, atypical spitzoid lesions, atypical cellular blue nevi, deep penetrating nevi, pigmented epithelioid melanocytomas etc. In fact, gene expression microarray hierarchical clustering maps will likely have the capacity to separate melanomas from nevi, identify different (histologically challenging) patterns of primary melanomas and clearly distinguish primary melanomas from sentinel node metastases. In preliminary studies the majority of differentially expressed genes (genes with the greatest fold-change between primaries and metastases) were genes that were decreased in metastases (S100A8, TACSTD2, SERPINB5, CLCA2, MMP1). Some genes were increased (MAGE family, PRKCB). Relatively increased keratinocyte-related genes in primary melanomas likely represent contamination of the tumor tissues by structures such as sweat ducts and glands. Information gained from studies of this type may provide understanding of the molecular events that underpin lymphatic invasion. In turn this will lead to recognition of the biomarkers that identify primary tumors with the potential for lymphatic extension.

Patients with melanoma have a predominant and early involvement of immunological dysfunctions affecting myeloid cells. Particularly, CD14+HLA-DR^neg/low ^representing bona fide myeloid derived suppressor cells (MDSC) in this tumor histology [8], accumulate in peripheral blood of melanoma patients since the very beginning of the disease (stage IIB and C) and can be detected as infiltrating components of primary lesions, suggesting a potential involvement of these cells in melanoma progression. CD14+HLA-DR^neg/low ^spontaneously release a large array of immunosuppressive and pro-tumorigenic cytokines and chemokines, and inhibit proliferation and function of activated T cells mostly through TGFβ secretion. Since patients with lower frequency of CD14+HLA-DR^neg/low ^and lower TGFβ serum levels mount better immune responses to anti-tumor vaccine [8], CD14+HLA-DR^neg/low ^down-modulation could be an opportunity to enhance immunotherapy. In this view studies are undergoing to identify potential pharmacological tools interfering with MDSC differentiation and function both in vitro and in vivo, in melanoma patients.

Cancer alters immune function via multiple mechanisms. To gain insights into the molecular mechanisms of immune dysfunction in cancer, gene expression profiles of peripheral blood lymphocytes (PBLs) from 12 patients with melanoma was compared to PBLs from 12 age-matched healthy controls. Of 25 significantly altered genes in T cells and B cells from melanoma patients, 20 were interferon (IFN)-stimulated genes (ISG). The functional response of lymphocytes to IFN stimulation was assessed by measurement of STAT1 phosphorylation (pSTAT1), an essential event in signal transduction by IFNs. The median percentage of phosphorylated STAT1-positive lymphocytes induced by IFN-stimulation was significantly reduced in patients with melanoma compared to healthy controls. In a subsequent study, it was shown that ISG expression is also reduced in PBLs from breast cancer patients. IFN-α-induced pSTAT1 is reduced in T cells, B cells and NK cells from breast cancer, melanoma and gastrointestinal cancer patients, while IFN-γ-induced pSTAT1 is reduced in B cells from all three cancer patient groups. Age is associated with decreased STAT1 responsiveness to IFN-α in melanoma.

These defects in IFN signaling are not influenced by chemotherapy, and the impairment in IFN signaling can be partially overcome by prolonged, high dose IFN-α. Moving beyond IFN signaling, three other JAK/STAT signaling pathways are downregulated and one pathway is upregulated in PBLs from melanoma patients. Thus, there appears to be global alterations in immune signaling networks necessitating use of Bayesian Network analysis to understand immune signaling networks in melanoma. Clinical application of these data led to the analysis of IFN signaling in lymphocytes from melanoma patients (stages IIIB or IIIC) pre- and post-HDI, and correlation with clinical response and outcome. Melanoma patients who had a clinical response to HDI therapy over the 4-week induction phase of neo-adjuvant therapy had a significant increase in the fold induction of pSTAT1 in peripheral blood T cells during IFN-stimulation from day 0 to day 29 and this correlated with good clinical outcome. Increase in pSTAT1 may be used to guide selection of patients for continued HDI therapy. The sample size of this study was too small (16 patients) to be conclusive, but these results indicate the need for a larger confirmatory study [[9,10], and Simons DL, Lee G, Kirkwood JM, and Lee PP. **Interferon Signaling Patterns in Peripheral Blood Lymphocytes may Predict Clinical Response and Outcome after High-Dose Interferon Therapy in Melanoma Patients**. *Submitted*.].

Some strategies augment vaccine efficacy, demonstrating that successful immunotherapy of melanoma will require interventions that reduce the number or function of Treg cells. Studies in mice suggest that vaccination of reconstituted lymphopenic hosts could elicit superior anti tumor immunity relative to normal hosts, highlighting the potential clinical benefit of performing tumor vaccination during immune reconstitution. However lymphopenic mice reconstituted with spleen cells from tumor-bearing mice (TBM) failed to generate tumor-specific T cells with therapeutic efficacy. Clinical trials in reconstituted lymphopenic patient showed that immediately following vaccination the absolute number of dividing Treg cells in peripheral blood is increased and the majority of Treg come from the reinfusion product. Therefore it was considered of interest to ex vivo deplete CD25+Treg from TBM spleen cells prior to reconstitution and vaccination: this strategy fully restored the generation of therapeutic effector T cells, even in animals with established tumor burden. Given these results a translational clinical trial in patients with metastatic melanoma has been initiated to exploit lymphopenia to augment the adoptive immunotherapy of melanoma patients. Preliminary studies of Helios protein expression in patients adoptively transferred with CD25-depleted PBMC and vaccinated following non myeloablative chemotherapy suggests that the majority of early recovering Treg are not thymus-derived. This suggests a critical role for the tumor milieu in promoting the recovery of Tregs. How can we interfere with the capacity of the tumor/tumor-bearing environment to generate tumor-induced Treg and promote the development of natural Treg? There are various options currently in study such as TGFβ blockade and anti-OX40 that can prevent generation of tumor-induced Treg in preclinical models. Another option is partial CD4 depletion that reduces Treg number and recovers tumor-specific and therapeutic T cell function in preclinical models. A number of these strategies are in clinical trials and combination studies that include vaccines are considered promising.

## Clinical Trials

Melanoma therapy has been difficult over the past 30 years, with many negative trials and the absence of any predictive markers for the few existing therapeutic agents. Adjuvant therapy of melanoma is the setting that may lend itself to the improvement of treatment given the series of studies of the ECOG and US Intergroup known as E1684, E1690, E1694, and the meta-analysis of all trials of IFN-α, which have confirmed a durable and significant impact of this therapy upon relapse-free and overall survival. Two approaches have been adopted to improve the relative magnitude and risk-benefit ratio for IFN-α: refine risk assessment, focusing treatment upon patients with greatest risk of relapse; and to refine therapeutic target, focusing treatment upon patients with greatest chance to benefit. For example, patients with high risk resected melanoma were studied to evaluate whether a high baseline or increasing serum S100B is an independent prognostic marker of risk for mortality. The studies [11] recently published concerning S100B have demonstrated that this marker allows us to refine the risk profile of melanoma and suggest that future studies of other risk biomarkers may add to our prognostic assessment of patients for adjuvant therapy. On the other hand, the appearance of autoantibodies or clinical manifestations of autoimmunity during treatment with interferon alfa-2b has been shown to be associated with statistically significant improvements in relapse-free survival and overall survival benefit of IFN therapy in patients with resected melanoma. Furthermore, baseline cytokine levels predict 5-year relapse-free survival with high-dose IFN-α. In conclusion profiling of sera from patients treated with HD-IFN identifies potential predictors of adjuvant therapeutic benefit. An unresolved question in adjuvant therapy with IFN-α is what the optimal duration of treatment may be. The results of the study E1697, which was designed to assess whether one month of IV high-dose 'induction' therapy is sufficient to improve relapse free and overall survival of intermediate and high-risk stage IIA and IIIA melanoma has been closed for futility in 2010. This demonstrates that one month of high-dose IFN is not sufficient for adjuvant therapy of high-risk patients, and argues that a year of therapy remains the standard of treatment. Multiple vaccine approaches, including the GSK DERMA phase III trial, are studied and are currently under study in adjuvant setting, but none has yet shown beneficial results. Novel melanoma vaccine strategies are being developed employing new CD8 killer T cell and CD4 helper T cell epitopes and utilizing polarized dendritic cells, (alphaDC1) loaded with melanoma peptides. However, the next chapter in melanoma therapy is likely to be comprised of the current active immunotherapy agents like IL-2 and IFN-α-2 with new immunotherapies such as the checkpoint inhibitors such as anti-CTLA4-blocking antibodies, and anti-PD1. After positive results in advanced disease, the adjuvant role of ipilimumab has been tested in two studies: EORTC18071 (in which it is compared to placebo) and ECOG E1609 in which it is compared to High-dose IFN. Ipilimumab is also being evaluated in a trial of neoadjuvant treatment that is nearing completion at the University of Pittsburgh.

A better understanding of the biology of melanoma is leading to the development of personalized treatment based on genetic alterations, molecular markers, risk classifiers and pharmacogenomics. Key studies with BRAF inhibitors are currently ongoing and more are starting. Despite profound responses, patients with BRAF mutant tumors eventually develop resistance and disease progression. Mechanisms of resistance are being identified, and studies are designed with different strategies to overcome this resistance. For example, new evidence suggests that both the MAPK and PI3K/AKT pathways can override BRAF inhibition and a combination blockade of both pathways after BRAF inhibitor failure will be tested in a randomized phase II of combined MEK inhibitor AZD6244 and AKT inhibitor MK2206 versus MEK inhibitor alone in patients with BRAF V600E mutant advanced unresectable melanoma who previously failed a selective BRAF inhibitor. cKIT is a target mainly in mucosal, acral and solar melanomas that can be targeted with inhibitors such as nilotinib and dasatinib. These are currently being studied in the phase III TEAM trial (nilotinib against dacarbazine in the treatment of metastatic and/or inoperable melanoma harboring a c-Kit mutation) and the phase II E2607 trial (dasatinib in patients with unresectable locally advanced or stage IV mucosal, acral and solar melanomas). The growing interest in the targeting of embryonic developmental pathways has led to the identification of Notch as a possible therapeutic target in melanoma. New molecules such as inhibitors of γ-secretase (GSI), a molecule involved in the activation of Notch signaling are currently in clinical development. RO4929097 is a GSI being studied in melanoma in a pilot biomarker-driven neoadjuvant study in resectable stage IIIB, IIIC, or IV, in a phase II trial as single agent in advanced unresectable or metastatic disease or in combination with chemotherapy (phase Ib/II trial of RO GSI in combination with cisplatin, vinblastine, and temozolomide in patients with metastatic melanoma). For immunotherapy, several studies are currently ongoing in advanced melanoma to refine the application of ipilimumab (dacarbazine and ipilimumab versus dacarbazine with placebo, bevacizumab plus ipilimumab, ipilimumab in patients with spontaneous preexisting immune response to NY-ESO-1, and study of BMS-908662, a Raf inhibitor, in combination with ipilimumab in subjects with advanced melanoma). Additional studies are starting to define the role of new molecules and novel combination treatments (dose-escalation study of combination BMS-936558, anti-PD1, and ipilimumab, biotherapy with aflibercept, VEGF-trap, and high dose IL-2 versus high dose IL-2 alone, anti-PD1 in combination with multiple class I peptide vaccines, or IL-12-based multipeptide vaccination with T-reg depletion).

Active immunotherapy approach have been developed this last decade, among which the clinical development of ASCI (Antigen-Specific Cancer Immunotherapeutic). This approach is aimed at educating the immune system to eradicate cancer cells by targeting specific antigens present on the tumors cells. MAGE-A3 antigen, one of these specific tumor antigens, is expressed by up to 76% of metastatic melanomas [12]. In a Phase I dose escalation study, patients with metastatic MAGE-A3 positive melanoma were immunized with recombinant MAGE-A3 protein associated with the immunostimulant AS02_B _to evaluate the safety profile and the clinical response following immunization. All dosage levels were well tolerated and no dose-toxicity relationship was observed. The clinical activity was mainly observed in early metastatic disease and no differences in immunogenicity were reported between different doses of protein tested (30, 100, 300 mg) [13]. Then a Phase II study was designed in patients with MAGE-A3 + cutaneous melanoma to evaluate MAGE-A3 recombinant protein combined with different immunostimulants (AS) AS15 or AS02_B _(NCT00086866). Results demonstrated that both MAGE-A3 ASCI formulations were well tolerated, recMAGE-A3 + AS15 seemed to be more active than recMAGE-A3 + AS02_B _and showed long-lasting clinical responses. The patients receiving recMAGE-A3 + AS15 also developed a more frequent and robust immune response. The main outcome of this study was the selection of the AS15 as adjuvant system for further development (Table 1) [14,15]. These results represent a second positive signal of clinical activity for the MAGE-A3 ASCI. Clinical activity was also reported in a separate double-blind, placebo-controlled Phase II study of patients with NSCLC (NCT00290355) (Table 2) [16]. Both Phase II trials in NSCLC and melanoma patients led to phase III trials initiation in melanoma (DERMA trial, resected MAGE-A3 + pIIIB/pIIIC melanoma randomized to recMAGE-A3 + AS15 or placebo - NCT00796445) and NSCLC (MAGRIT trial, resected MAGE-A3+ NSCLC pIB/II/IIIA randomized to recMAGE-A3 + AS15 or placebo with or without prior chemotherapy - NCT00480025) to show the efficacy of the MAGE-A3 ASCI. Moreover, gene profiling of melanoma tumors taken prior to MAGE-A3 ASCI immunization has led to the identification of a gene signature (GS) that may predict the clinical outcomes of MAGE-A3 ASCI treatment. Most of the genes identified in the GS were immune-related suggesting that the presence of a specific tumor-environment prior to MAGE-A3 ASCI treatment influences its efficacy. The predictive value of the melanoma signature was also tested in NSCLC and showed that patients with the GS are more likely, but not certain, to benefit from MAGE-A3 ASCI immunization (Table 3) [17]. The GS is currently under validation.

**Table 1:** Results from Phase II study in cutaneous metastatic melanoma [14,15]

Phase II Melanoma (NCT 00086866)		recMAGE-A3 + AS02_B_	recMAGE-A3 + AS15
**Primary endpoint**	**Clinical objective responses**	- **1 PR (5-months)** **5 SD (> 16 weeks)**	**3 CR (11, 32+, 23+ months)** **1 PR (7-months)** **5 SD (> 16 weeks)**

Secondary endpoints	Safety	Well tolerated	Well tolerated

	Overall survival	19.9 month (95% CI: 15.4°; 25.6)	31.1 months (95% CI: 20.0°; NR)

	Cellular immune response	Induced in 21% of patients	Induced in 76% of patients

CR: Complete Response

PR: Partial Response

SD: Stable Disease

CI: Confidence Interval

NR: Not Reached

**Table 2:** Results from Phase II clinical study in Non-Small Cell Lung Cancer (NSCLC) with a median follow-up time of 44-months [16]

Phase II NSCLC (NCT 00290355)		Results recMAGE-A3 + AS02_B_
**Primary endpoint**	**DFI**	**HR: 0.75 (95% CI: 0.46-1.23)** **recMAGE-A3 + AS02_B _*vs*. Placebo** ***p *****= 0.127 in favor of MAGE-A3 ASCI**

Secondary endpoints	Safety	Well tolerated

	Humoral immune response	Response induced in > 98% patients

	Cellular immune response	Response induced in 41% patients

DFI: disease Free Interval

HR: Hazard Ratio

CI: Confidence Interval

NSCLC: Non-Small Cell Lung Cancer

**Table 3:** Gene signature associated to clinical benefit of MAGE-A3 ASCI: Identification in Phase II study in melanoma patients and confirmation in NSCLC patients [17]

	Phase II studies evaluating the MAGE-A3 ASCI	
	
	Phase II NSCLC (NCT 00290355)	Phase II Melanoma (NCT 00086866)
GS-	25% relative improvement (DFI)	OS of 16.2 months

GS+	53% relative improvement (DFI)	OS of 28.0 months

GS-: Population in which the gene signature was not found

GS+: Population for which a specific Gene Signature has been defined

DFI: Disease Free Interval

OS: Overall Survival

The importance of immunostimulatory antibodies in melanoma treatment was demonstrated in clinical trials of anti-CTLA-4. Two anti-CTLA-4 antibodies, ipilimumab and tremelimumab, are in clinical development, both of which block a key co-inhibitory signal mediated by the CTLA-4 receptor that regulates T-cell activation. A randomized phase III trial of ipilimumab 3 mg/kg every 21 days × 4 doses, compared to the gp-100 peptide vaccine or the combination of the two in previously treated metastatic melanoma patients, showed a significant improvement in overall survival for both ipilimumab arms over peptide vaccine alone. The results of the trial will likely result in regulatory approval of ipilimumab as a single agent. The most common adverse events were immune-related and included rash, diarrhea, endocrinopathies and hepatitis. Adverse events were almost always reversible and manageable with immunosuppressive medications. Development of immune related adverse events was associated but not necessary for response in some trials. Promising data are emerging from trials of anti-CTLA-4 in combination with other immunomodulatory agents, for example, in a phase 1/2 of IL-2 + ipilimumab and a phase II of tremelimumab + high-dose Interferon-α. Because of results from the anti-CTLA-4 trials, there is growing interest in developing other antibodies targeting T-cell co-stimulatory and co-inhibitory molecules. One of these, B7-H1, a recently described member of the B7 family of costimulatory molecules, is thought to be involved in the negative regulation of cellular and humoral immune responses through the PD-1 receptor on activated T and B cells. Expression of B7-H1 on mouse P815 tumor blocks the potent anti-tumor effects generated by tumor expression of the strong co-stimulatory signal B7.1. In the first single-dose phase I clinical trial of PD-1 blockade with the mAb MDX-1106, patients with advanced treatment refractory solid tumors were treated in dose-escalating six-patient cohorts at 0.3, 1, 3, or 10 mg/kg, followed by a 15-patient expansion cohort at 10 mg/kg. Anti-PD-1 was well tolerated with only one serious adverse event, inflammatory colitis, and produced one partial response and one mixed response among 10 metastatic melanoma patients. A second phase I trial evaluated the safety and antitumor activity of MDX-1106 administered every two weeks at doses of 1, 3, or 10 mg/kg. An MTD was not reached, and subsequently up to a total of 16 patients with metastatic melanoma were accrued at each of the three dose levels. Treatment was well tolerated. Serious adverse events were rare and included hepatitis, hypophysitis, hypersensitivity reaction, elevated lipase and colitis. Among 46 evaluable melanoma patients at the time of analysis, 15 partial responses were observed, all ongoing at a minimum follow up of 5+ months, confirming the safety and antitumor activity of MDX-1106. Based on the promising clinical results and supporting preclinical data, additional studies are under consideration, including combinations of anti PD1 and anti CTLA-4 mAb, or anti-PD1 with IL2 or IFN.

Evidence of clinical benefit of vaccination in patients with melanoma was given in a presentation of a phase III multi-institutional randomized study of immunization with gp100:209-217(210 M) peptide followed by high dose IL-2 vs. high dose IL-2 alone in patients with metastatic melanoma. Previously a phase II study showed objective responses in 42% of patients with metastatic melanoma receiving high-dose (HD) IL-2 plus gp100 peptide. Other studies showed a lower response rate (RR) but no randomized studies had been done. A prospective randomized (1:1) phase III trial was conducted at 21 centers enrolling 185 patients with stage IV or locally advanced stage III cutaneous melanoma, HLA A0201, no brain metastases, eligible for HD IL-2, no previous HD IL-2 or gp100 and ECOG 0 or 1. Arm 1 received HD IL-2 alone (720,000 IU/kg/dose) and Arm 2 gp100:209-217(210 M) peptide + Montanide ISA-51 each cycle followed by HD IL-2. The primary objective was to compare clinical response of HD IL-2 with and without gp100 vaccine. Secondary objectives were to evaluate toxicity, progression free survival, immunologic response and quality of life. Central HLA typing, pathology review, and blinded response assessment were done at the NIH. From 2000 to 2007 185 patients were enrolled and 93 were treated in Arm1 and 86 in Arm 2. Pretreatment patient characteristics were well balanced except for a trend of younger patients in the vaccine arm. Toxicities were consistent with HD IL-2 ± vaccine, and manageable with medications. Investigator and central response assessment showed significant improvement in overall RR and progression free survival for Arm 2. Patients with lung metastases (M1b) accounted for the majority of the response difference. A trend for increased overall survival with gp100 vaccine was observed, with a median overall survival in Arm 2 of 17.6 months versus 12.8 in Arm 1. Median follow up for surviving patients was 41.5 months. In a 12 day in-vitro sensitization assay of PBMC, the level of vaccination was low (19%) and did not correlate with clinical response, confirming previous studies. Increased T regulatory cells (CD4+foxp3+) in responders after 4 cycles of treatment was seen in both treatment arms. In conclusion gp100 enhanced the clinical activity of HD IL-2 in patients with metastatic melanoma. Rational combinations of vaccines and immunomodulatory agents like IL-2 need to be further studied in the treatment of patients with metastatic melanoma [18].

Alterations in chromatin structure profoundly influence gene expression during normal cellular homeostasis and malignant transformation. Methylation of cytosines within CpG islands located in promoter and proximal coding regions facilitates recruitment of chromatin-remodeling proteins, which inhibit gene expression. Post-translational modifications, such as acetylation, methylation, and phosphorylation, of core histone proteins ''mark'' regions of chromatin for recognition by multiprotein complexes, which either promote chromatin relaxation and gene expression, or chromatin compaction and repression of gene expression. Epigenetic modification are reversible pharmacologically and exploitable for the development of more efficacious immunotherapeutic regimens. Along this line the potential of the DNA hypomethylating drug, 5-aza-2'-deoxycytidine (5-AZA-CdR), to modulate the expression of cancer testis antigens (CTA) and of HLA class I antigens by melanoma xenografts, and the resulting modifications in immunogenicity of neoplastic cells was investigated. Molecular analyses demonstrated a de novo, long-lasting expression of the CTA MAGE-1, -2, -3, -4, -10, GAGE 1-6, NY-ESO-1, and the upregulation of the constitutive levels of MAGE-1, MAGE-3, and NY-ESO-1 in melanoma xenografts from 5-AZA-CdR-treated mice. Serological and biochemical analyses identified a de novo expression of NY-ESO-1 protein and a concomitant and persistent upregulation of HLA class I antigens. It was also observed that the generation of anti-NY-ESO-1 antibodies in Balb/C mice immunized with 5-AZA-CdR-treated human melanoma cells. In addition, treatment with 5-AZA-CdR induced a persistent expression of MAGE-1 in melanoma cells, and significantly enhanced the constitutive expression of HLA class I antigens and of the costimulatory molecules on a panel of melanoma cells. Altogether, the data obtained identify an immunomodulatory activity of 5-AZA-CdR in vivo and strongly support the design of novel strategies for clinical CTA-based chemo-immunotherapy for melanoma patients.

## New Molecules And New Strategies

After the failure of sorafenib, other more selective inhibitors that target the mutated BRAF kinase have been developed and are currently being evaluated in clinical trials. At the moment, the two that appear to be the best BRAF inhibitors have been tested in clinical studies: PLX4032 and GSK2118436. The phase I trials of both agents were described and their toxicities and efficacy were compared. The most frequent adverse event have been rash (68%), arthralgia (48%), photosensitivity (42%), and fatigue (32%) for PLX4032 and pyrexia (43%), rush (30%) and headache (26%) for GSK2118436. A characteristic toxicity of these drugs is the onset of cutaneous squamous cell carcinoma (23% for PLX4032 and 7% for GSK2118436) which was suggested to be at least in part due to inhibition of wild-type BRAF kinase and enhanced signaling through RAF 1 signaling. At the maximum tolerated dose of 960 mg twice daily of PLX4032 tumor responses were rapid, with onset seen as early as 2 weeks of treatment by positron emission tomography scan, and an 81% best overall response rate is reported. In the phase II study treatment with PLX4032 resulted in a progression free survival of 6.2 months showing significant tumor shrinkage in the majority of patients (objective response rate occurred in 52% of patients) and median overall survival has not been reached. For GSK2118436 phase II expansion at 150 mg BID has shown overall response rate of 77% and responses were seen in many sites including brain. The rapid emergence of drug resistance in some patients treated with one of the other BRAF inhibitor highlights the need to establish mechanisms resistance in order to develop therapeutic strategies for overcoming or preventing resistance. Mechanisms of primary resistance based on alteration in MAP kinase signaling can potentially be overcame by combined BRAF and MEK inhibition based on preclinical evidence, and is being studied in a dose-escalation, phase IB/II study to investigate the safety, pharmacokinetics, pharmacodynamics and clinical activity of the BRAF inhibitor GSK2118436 in combination with the MEK Inhibitor GSK1120212 in subjects with BRAF mutant metastatic melanoma. Preliminary investigations into mechanisms of secondary resistance in patients treated with PLX4032 have suggested PDGFRβ or IGFR expression, emergence of NRAS mutation, and upregulation of COT/TPL2 suggest that targeting these molecules in combination with BRAF may extend the benefit of this approach. Preliminary evidence suggests that oncogenic BRAF contributes to immune escape and that blocking its activity via MAPK pathway inhibition leads to increased expression of melanocyte differentiation antigens whose recognition of is a critical component of the immunologic response to melanoma. However, treatment with MEK inhibitors impairs T lymphocyte function, whereas T-cell function is preserved after treatment with a specific inhibitor of BRAF. Thus, combinations of BRAF inhibitors with immunotherapy may be another rational direction to pursue.

These findings have important implications for combined kinase-targeted therapy plus immunotherapy for melanoma. In fact, various possible approaches for combined therapy of advanced melanoma were described. Recent data from trials testing targeted agents or immune modulators, showed an improved survival with ipilimumab and efficacy in inhibition of mutated, activated BRAF that will lead to new strategies of treatment. These includes target agents as BRAF-inhibitor with greater selectivity, MEK inhibitors that show efficacy in both BRAF mutated and NRAS mutated patient and c-Kit inhibitors in patients with c-kit mutated melanoma. Although response rates with these molecules are high, most are not durable due to the development of resistance to treatment. For example PTEN loss and cyclinD1 amplification are important regulators of intrinsic resistance to BRAF inhibitors. Combined PI3K and BRAF inhibitors therapy could help to overcome resistance. For immune modulators, among those that block immune checkpoint, in addition to anti-CTLA-4, anti-PD-1 also showed responses in metastatic melanoma patients and, furthermore, the combination of both in sub-efficacious doses demonstrated efficacy in a mouse model. Positive results are also emerging from studies of immune modulators that stimulate immune system as anti-CD137, anti-OX40, anti-CD40, anti TGF beta and anti 1-MT. Based on preclinical study findings, new possible targets for melanoma therapy are Notch, involved in embryonic pathways, NF-kB, given its dominance in the regulation of growth signals and in the immune and inflammatory response and PI3K inhibitors, that, already used in phase I clinical trial, demonstrated significant antitumor activities in breast cancer.

Other fields of cancer immunotherapy that are proving fruitful are oncolitic immunotherapy, which is an example OncoVEX^GM-CSF^and new immune modulators antibodies as Darleukine, a fusion protein, consisting of the human vascular targeting antibody L19 and of human interleukin-2. The next challenge for melanoma therapy will be, from the gene signature, to optimize the treatment of individual patient using these active agents sequentially or in combination each other or with traditional anticancer modalities such as chemotherapy, radiation or surgery [19].

The adaptive immune response influences the behavior of human tumors. In fact, characterization of the tumor-infiltrating immune cells in large cohorts of human colorectal cancers by gene expression profiling and in situ immunohistochemical staining to evaluate the expression levels of genes related to inflammation, TH1 adaptive immunity, and immunosuppression suggested that TH1 adaptive immunity has a beneficial effect on clinical outcome [20]. Tissue microarrays to investigate the in situ adaptive immune response in the center of the tumor (CT) and the invasive margin (IM) of 415 CRCs showed that tumors from patients without recurrence had higher immune cell densities (CD3, CD8, GZMB, and CD45RO) within each tumor region (CT and IM), than did those from patients whose tumors had recurred. For all the markers of the combined analysis of CT plus IM regions demonstrated that coordinated adaptive immune reaction more than tumor invasion predicts clinical outcome. Collectively, the immunological data (the type, density, and location of immune cells within the tumor samples) were found to be a better predictor of patient survival than the histopathological methods currently used to stage colorectal cancer [21,22]. Hence the concept of ''immune contexture'' as the combination of immune variables associating the nature, density, functional orientation and distribution of immune cells within the tumor of a natural in situ immune reaction [23,24]. In order to understand the mechanisms underlying immune responses in colorectal cancer data integration and biomolecular network reconstruction are applied. The presence of specific chemokines (CX3CL1, CXCL10, CXCL9) correlate with high densities of T-cell subpopulations within specific tumor regions and their high expression with prolonged disease-free survival [25]. According to an immune score based on the evaluation of CD45RO-CT/IM and CD8-CT/IM the prognostic significance of immune criteria was compared with that of the tumor extension criteria using the American Joint Committee on Cancer/International Union Against Cancer-TNM (AJCC/UICC-TNM) staging system. Assessment of CD8^+ ^cytotoxic T lymphocytes in combined tumor regions provides an indicator of tumor recurrence beyond that predicted by AJCC/UICC-TNM staging [21,22,26]. Similarly there is a correlation between the extent of immune cell density, tumor stage and relapse in melanoma [27]. In addiction for most of the malignancies is demonstrated over the time a correlation between lymphocytic infiltration and survival benefit for patients with cancers [24,28]. These findings, though a revision of the current indicators of clinical outcome, may help to better identify the high-risk patients who would benefit from adjuvant therapy.

Since T lymphocytes mediate durable tumor regressions after immunotherapy, TCR engineering adoptive cell transfer strategies is devised to take the TCR genes from one subject who rejected melanoma and use them to engineer a melanoma-fighting immune system in other subjects. Adoptive cell transfer (ATC) of splenocytes from fully immunocompetent HLA-A2.1/Kb mice transduced with a chimeric murine/human TCR specific for tyrosinase (MART-1), together with lymphodepletion conditioning, dendritic cell-based vaccination, and high dose interleukin-2, had profound antitumor activity against large established MHC-and antigen-matched tumors. Genetic labeling with bioluminescence imaging and positron emitting tomography reporter genes allowed visualization of the distribution and antigen-specific tumor homing of TCR transgenic T cells, with trafficking correlated with antitumor efficacy. This approach, directly translatable to humans, lead to developed of a study of gene modified immune cells in which adoptive transfer of MART-1 F5 TCR engineered peripheral blood mononuclear cells (F5) after a nonmyeloablative conditioning regimen, are administrated with of MART-126·35-pulsed dendritic cells and interleukin-2, in patients with advanced melanoma. Early results showed that the treatment involves hematological toxicity (neutropenic fevers, marrow aplasia) and transient responses followed by progression. One of the potential mechanisms of relapse is the decrease in frequency of TCR transgenic cells after ACT. In addition the application of a nanotechnology-based diagnostics as NACS (Nucleic Acid Cell Sorting) and SCBC (Single Cell Barcode Chip) allowed evaluating the functional heterogeneity of MART-1 cytotoxic T lymphocytes of the same patient and its relation with temporal profile of disease. Next clinical trials are planned of association of F5 with tremelimumab, NY ESO TCR, or vorinostat.

Most human melanomas contain tumor-reactive T-cells. Using IL-2 these can be activated and grown in vitro and than can be transferred back to a suitably prepared patient along with systemic IL-2 to circumvent the limited ability of vaccines to generate CTL. Objective clinical responses have been observed in patients who received non-myeloablative chemotherapy prior to the adoptive transfer of autologous melanoma-reactive tumor-infiltrating lymphocytes (TILs) and successes have also been achieved in the treatment of small brain metastases. Indeed ACT with a nonmyeloablative preparative regimen using TILs and interleukin-2 has demonstrated to mediate complete and durable regression of melanoma brain metastases. Disadvantages of this approach are that, since the assay for tumor recognition is not perfect, some active TILs are discarded inappropriately and selecting tumor reactive cultures complicates and prolongs TILs growth making labor-intensive TILs production and limiting its widespread applicability. Therefore short-term TILs cultures have been developed for which demonstrating tumor recognition is not required. These ''young TILs'' can mediate tumor regression in metastatic melanoma with manageable toxicity constituting the next chapter of ACT therapy. Returning to the concept of TCR gene therapy, it was shown that retroviral insertion of genes encoding tumor-reactive TCRs can impart tumor recognition, providing the means for generating potent Ag complex-specific TCR genes for T cell adoptive immunotherapy. This approach has proven effective against NY-ESO1 in melanoma and synovial sarcoma patients and in at least one patient targeting CEA in colorectal adenocarcinoma. Genetically retargeting T-cells by inserting antibody-based chimeric antigen receptors (e.g. anti CD19 and anti VEGF-R2) or introducing other immunological functions (such as "single chain" IL-12 secretion) are also promising. Still, one of the main future directions of research will remain identifying highly specific new tumor target antigens to attack with these new technologies.

## Competing interests

PAA participated in advisory board for Bristol Myers Squibb, Merck/Schering-Plough, GlaxoSmithKline and Roche. MKS has served on the Merck/Schering-Plough Advisory Board and Speakers' Bureau, and owns stock in Melanoma Diagnostics. Myriad Genetics has licensed intellectual property developed by MKS. JMK is consultant to GSKbio and Morphotek. FFL is employee of GlaxoSmithKline Biologicals. MS received consulting fees from Bristol Myers Squibb. KF is consultant to Roche/Genentech and GlaxoSmithKline. AR participated in advisory board for Roche-Genentech and Bristol Myers Squibb.

The other authors have no competing interests to declare.

## Authors' contributions

PA, EDM, and FMM prepared the manuscript collaboratively with input and review by all co-authors. All Authors read and approved the final manuscript
